# Supplementary figures and images for: Association of colitis with gut-microbiota dysbiosis in clathrin adapter AP-1B knockout mice
Source: PLoS One. 2020 Mar 24;15(3):e0228358. doi: 10.1371/journal.pone.0228358 (PMC7093000; doi:10.1371/journal.pone.0228358)

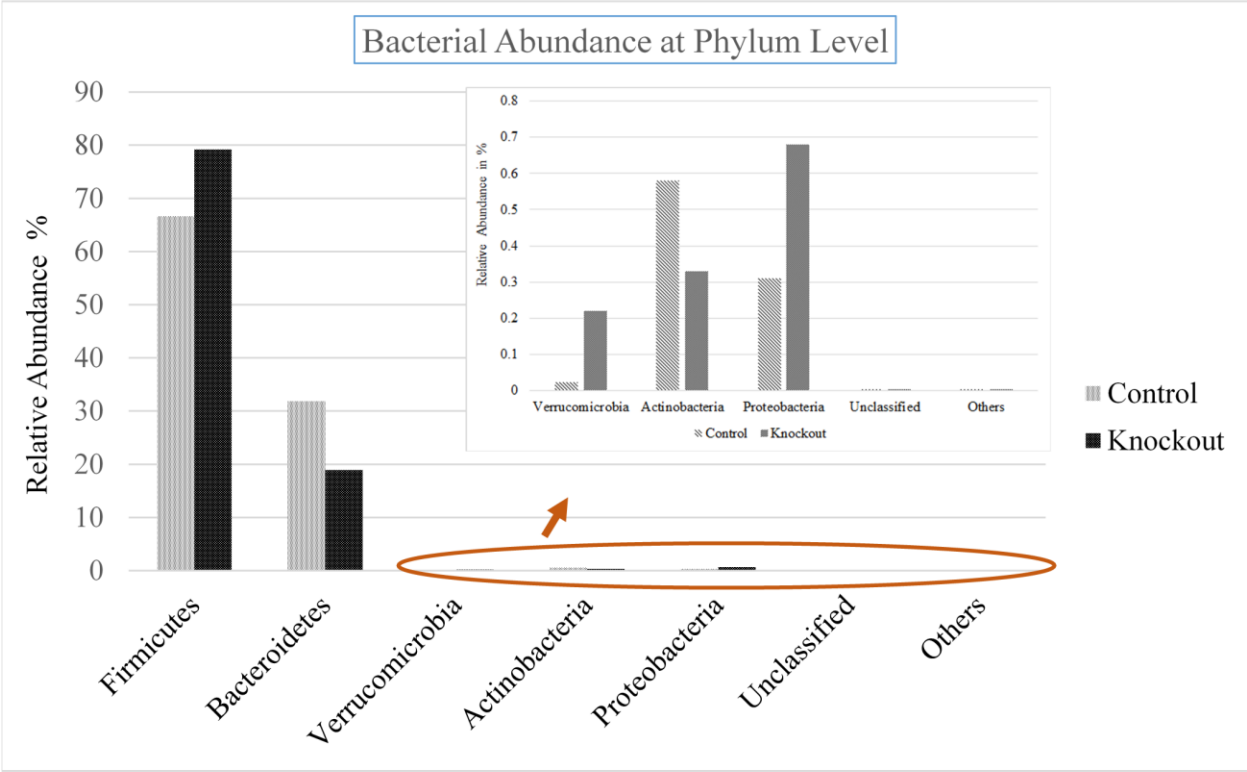

Supplement: S1 File — The inset shows the relative abundance on a different scale for the phyla Verrucomicrobia, Actinobacteria, Proteobacteria, and others. (PDF) [file pone.0228358.s001.pdf]

Figure A

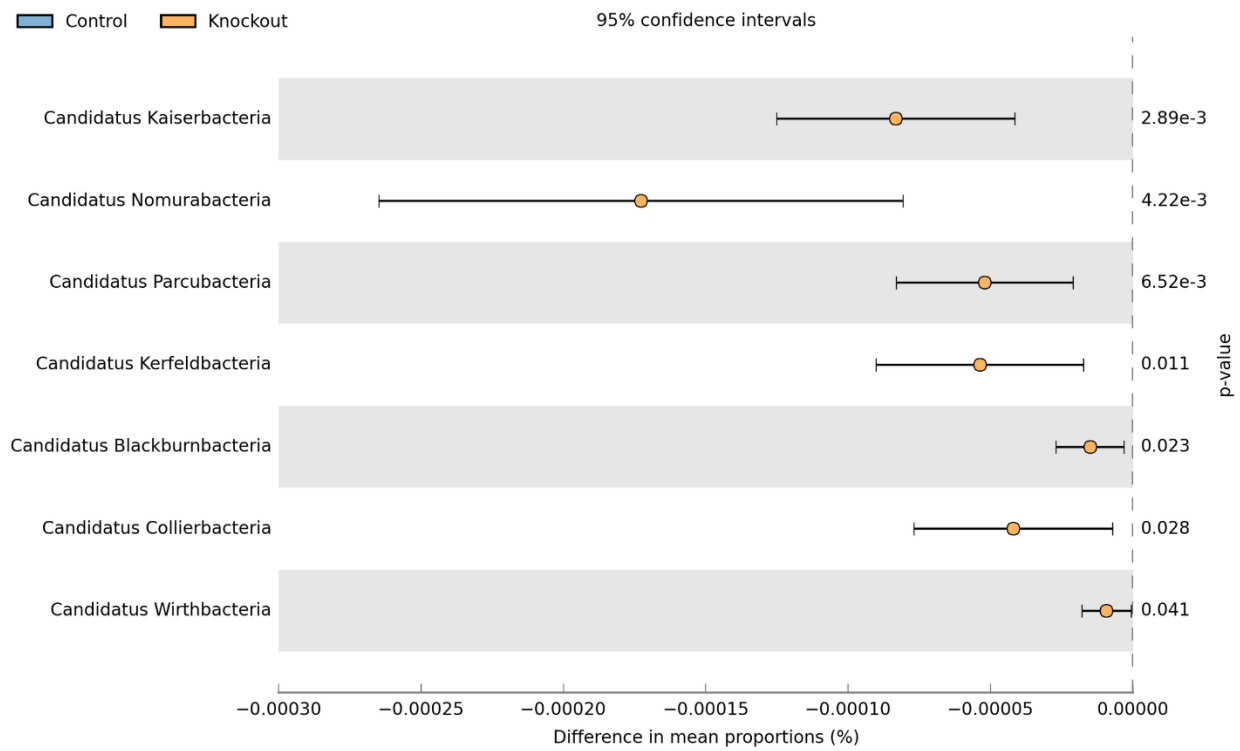

Figure B

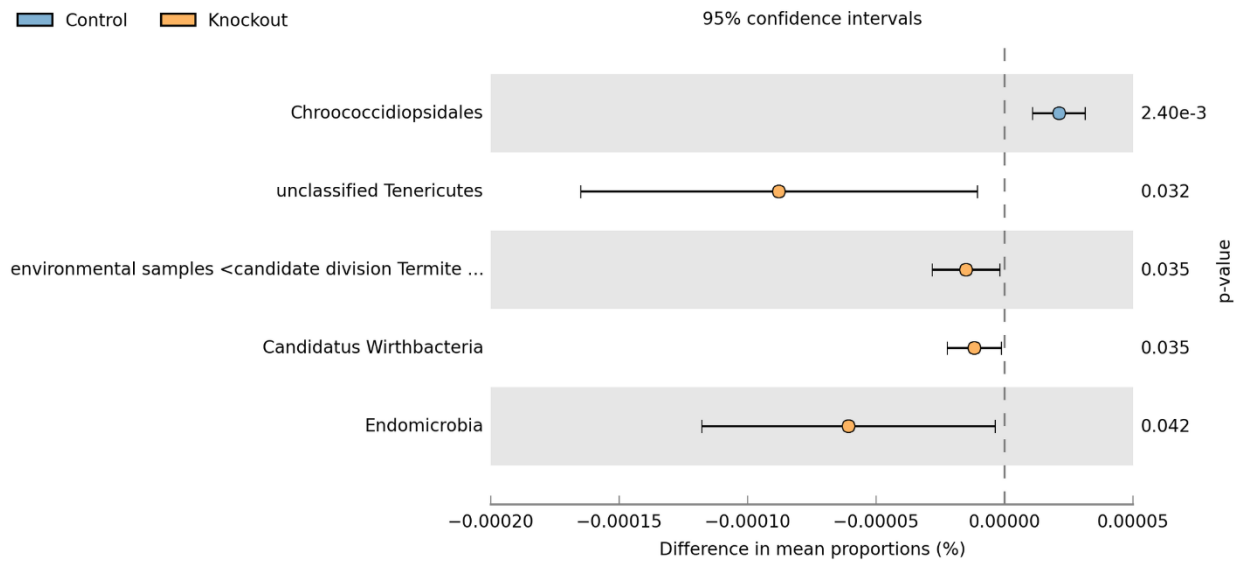

Figure C

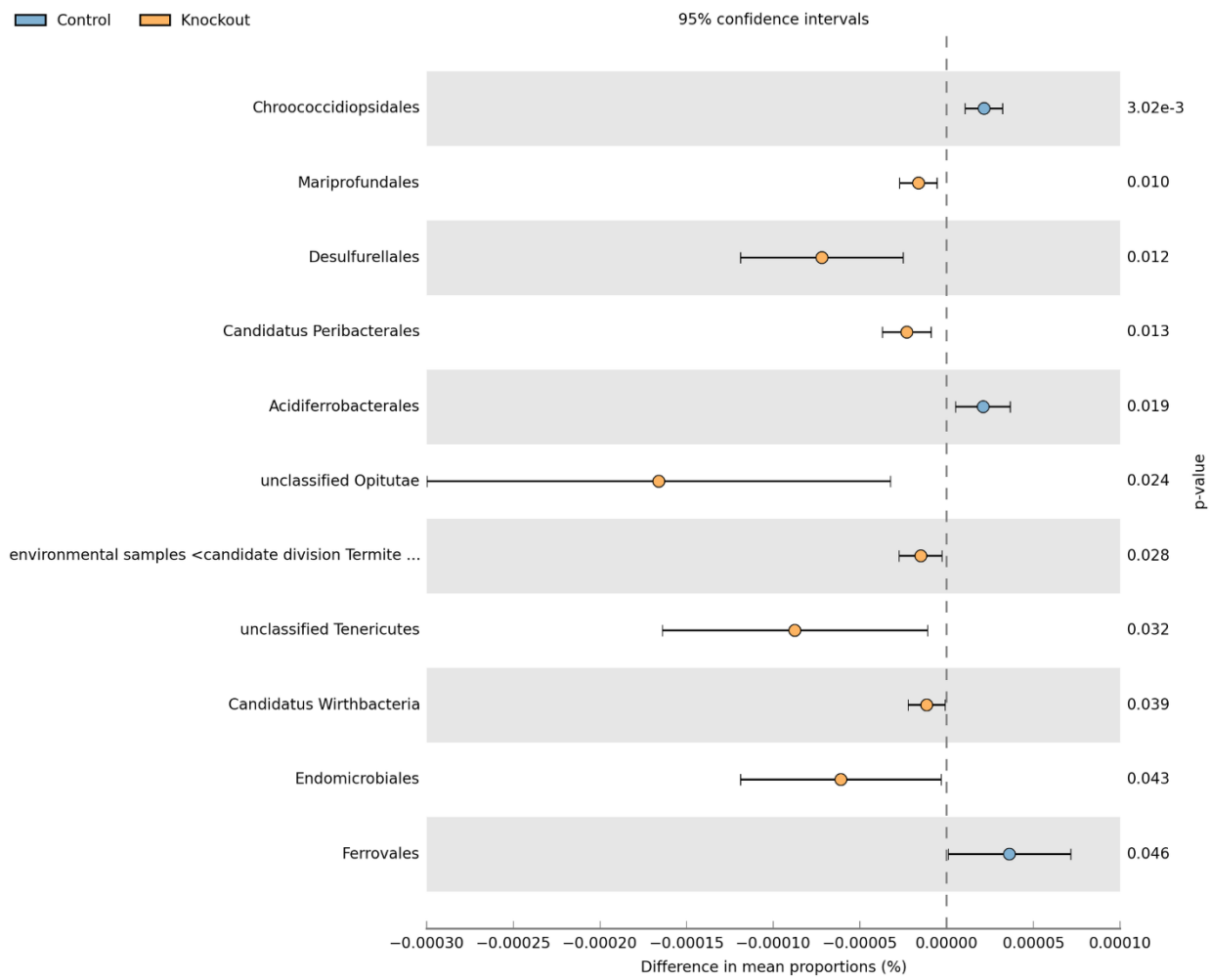

Figure D

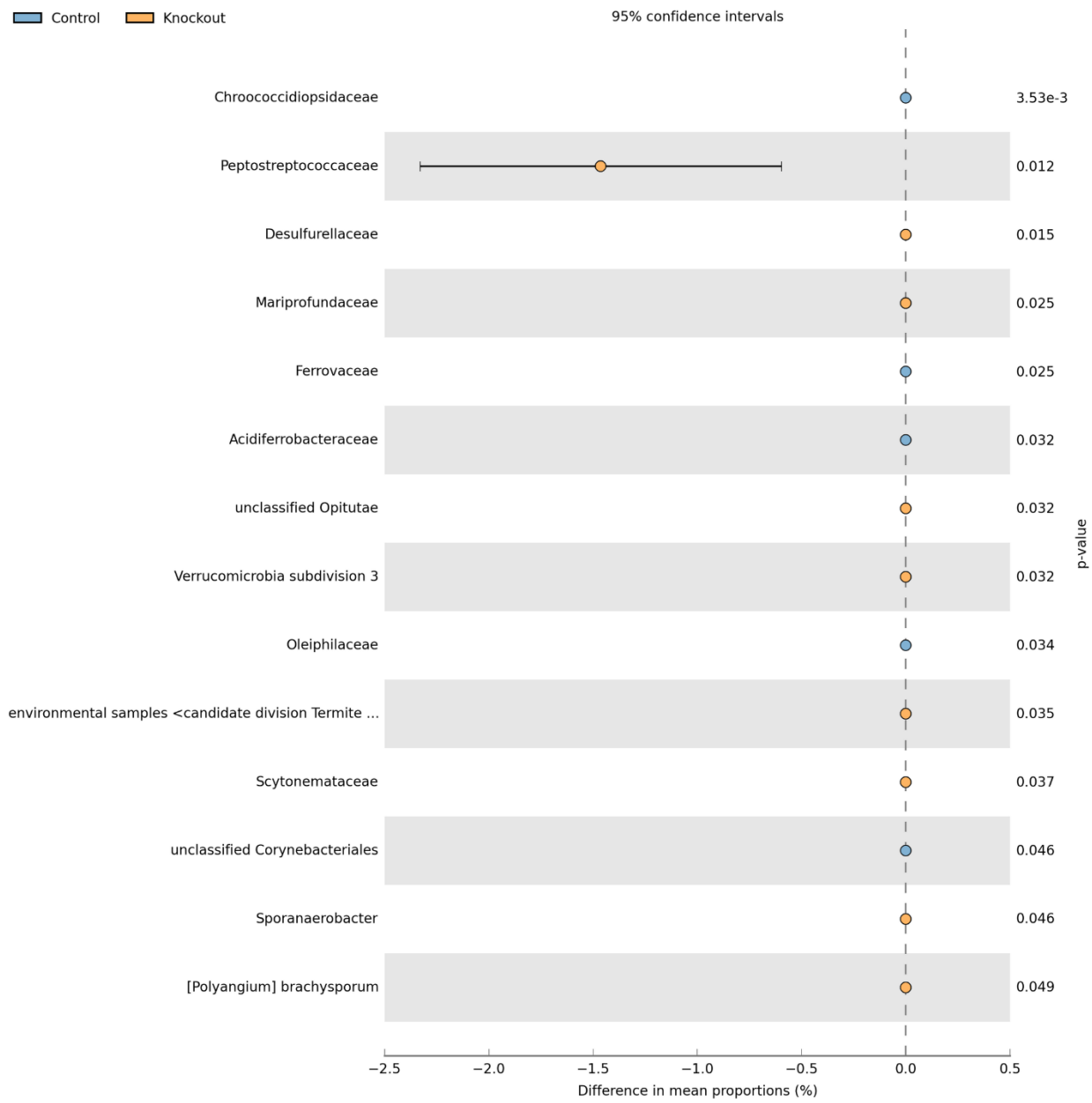

Figure E

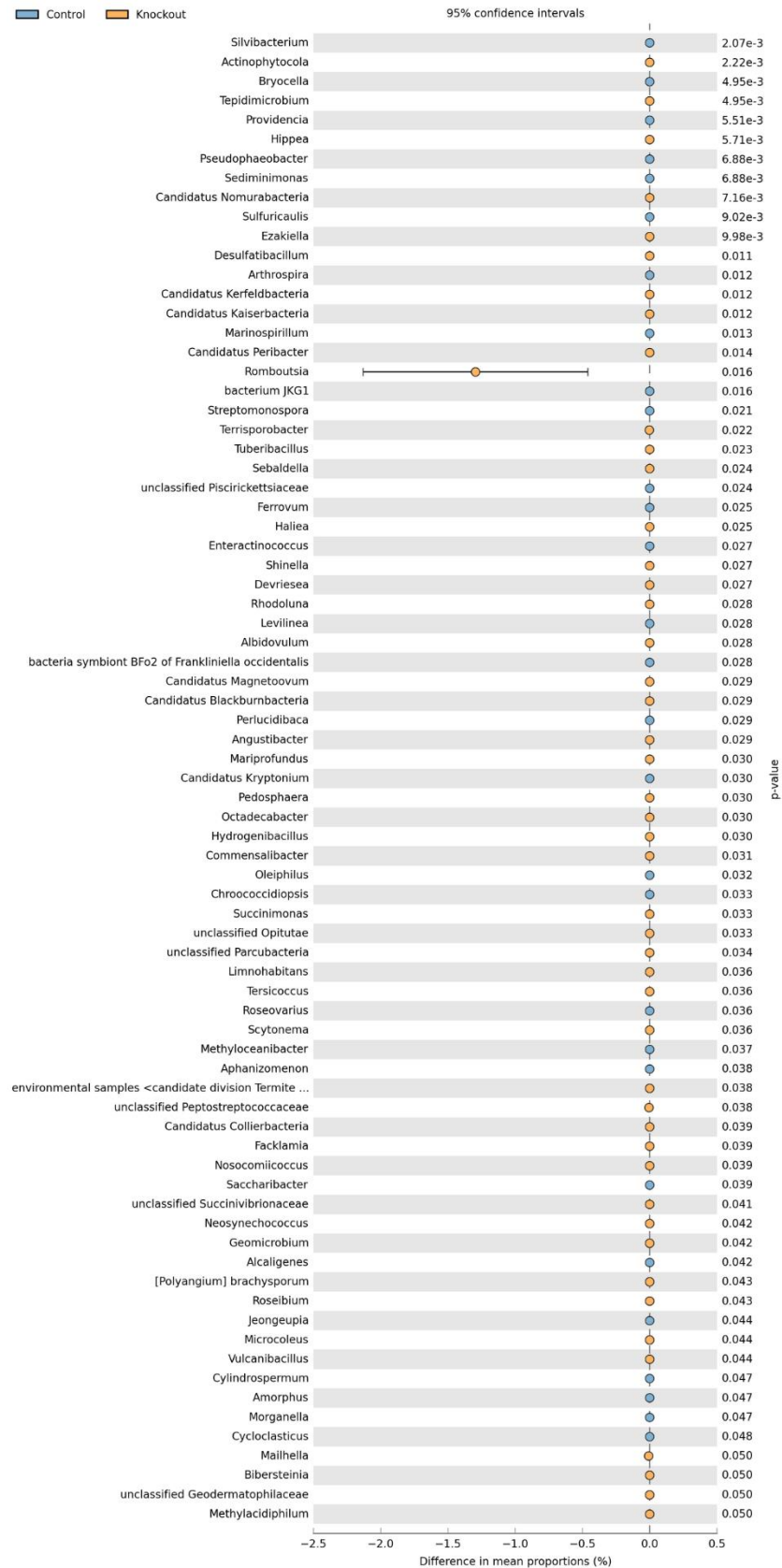

Supplement: S2 File — Extended error bar chart representing the differences in the mean proportions between the CO (blue) and KO (orange) groups at the level of (A) phylum, (B) class, (C) order, (D) family, and (E) genus. Statistical significance was tested using the Welch’s t-test with p < 0.05 (PDF) [file pone.0228358.s002.pdf]

Figure A

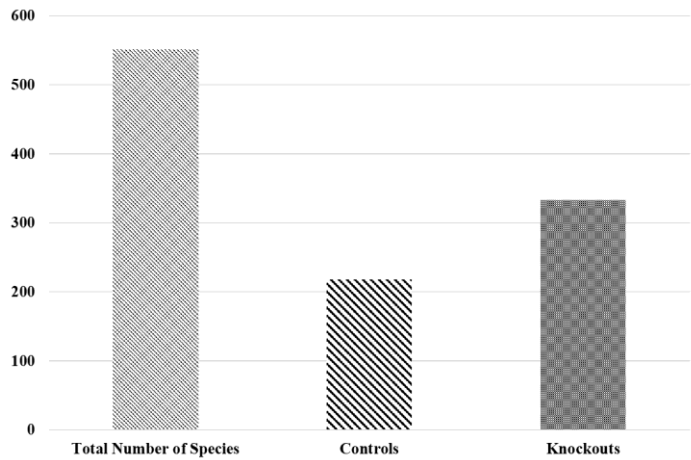

Figure B

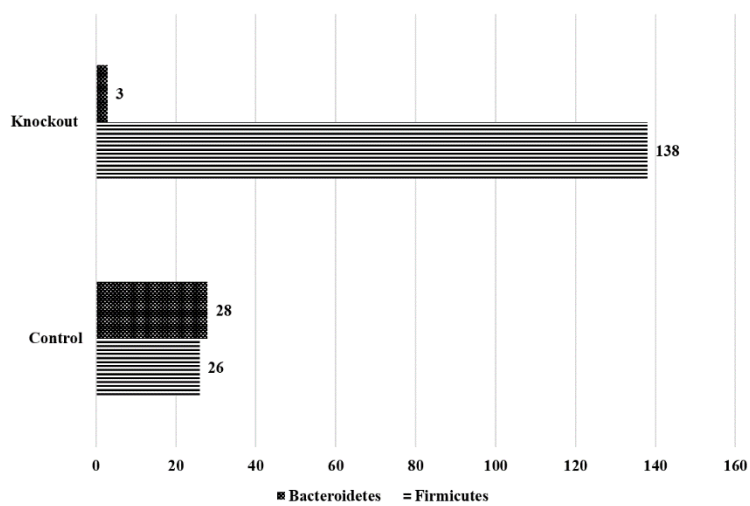

Supplement: S3 File — (A) Distribution of 551 species, (B) Distribution of the number of species belonging to the phyla Firmicutes and Bacteroidetes in the CO and KO Groups. (PDF) [file pone.0228358.s003.pdf]

Figure A

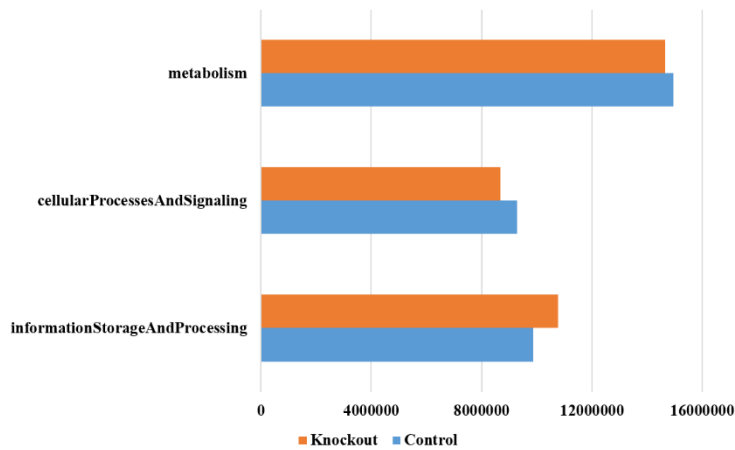

Figure B

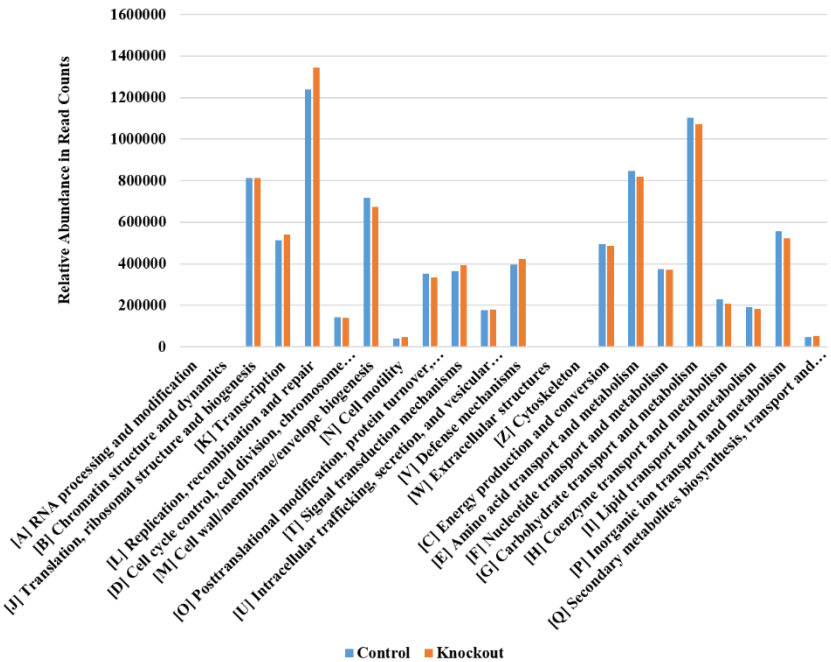

Figure C

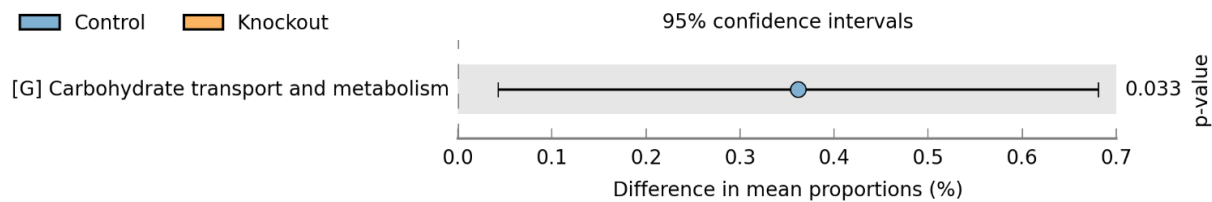

Supplement: S4 File — Distribution of the number of metagenomic reads in the CO and KO groups (A) in the three functional classes at level 1, (B) in different functional classes at level 2 of EggNOG-based classification. (C) Extended error bar plot representing the differences in the mean proportions of the functional category “carbohydrate transport & metabolism” between the CO and KO groups. Statistical significance was tested using the Welch’s t-test with p < 0.05. (PDF) [file pone.0228358.s004.pdf]
