# Supplementary material for: Association of colitis with gut-microbiota dysbiosis in clathrin adapter AP-1B knockout mice
Source: PLoS One. 2020 Mar 24;15(3):e0228358. doi: 10.1371/journal.pone.0228358 (PMC7093000; doi:10.1371/journal.pone.0228358)
Supplement: Attachment — Submitted filename: Response to Reviewers.DOCX [file pone.0228358.s014.DOCX]

**Rebuttal Letter:**

**Reviewer #1:** We think that the manuscript still needs careful editing to correct for several mistakes. Just to take 2 examples, in the new manuscript (with modifications indicated in the text), the correct name for H2S is hydrogen sulfide, not hydrogen sulfate (line 215), and on line 340, the sentence "... hydrogen sulfide (H2S) is a phenotype of colitis" is unclear. Do you mean "Excessive hydrogen sulfide in the intestinal luminal content may participate to colitis" ? Please correct with the relevant literature.

Reply: We thank you for giving us an opportunity to revise our manuscript. In the manuscript with track changes mode on, the following suggestions has been incorporated:

**Page no. 9; line no. 204-206: Modified “**Sulfate-reducing bacteria have been suggested to aggravate gastrointestinal diseases by making hydrogen sulfate (H_2_S) and other harmful by-products as well as by reducing beneficial metabolites, such as butyrate” **🡪 “**Sulfate-reducing bacteria have been suggested to aggravate gastrointestinal diseases by making hydrogen sulfide (H_2_S) and other harmful by-products as well as by reducing beneficial metabolites, such as butyrate”

**Page no. 16; line no. 311-312: Modified “**It has been reported previously [29] that hydrogen sulfide (H_2_S) is a phenotype of colitis**”🡪 “**It has been reported previously that an excessive hydrogen sulfide (H_2_S) production is found in the feces of ulcerative colitis patients [29].**”**

The reference for the above statement is already included in the manuscript.

**Page no. 18; line no. 342-344: Modified “**In addition, H_2_S, a cytotoxic byproduct of SRB that exerts pro-inflammatory effects at high concentrations [45, 46], can exacerbate intestinal epithelial barrier damage and is a colitis phenotype.**”🡪 “**In addition, H_2_S, a cytotoxic byproduct of SRB that exerts pro-inflammatory effects at high concentrations [45, 46], can exacerbate intestinal epithelial barrier damage and its excessive production is reported in colitis condition [26].**”**

In the above statement we have included reference [26] in the revised manuscript.

Apart from these changes, some other grammatical mistakes and typographical errors were also corrected as marked in the revised manuscript.
